# Metasurfaces for Far-Field Radiation Pattern Correction of Antennas under Dielectric Seamed-Radomes

**DOI:** 10.3390/ma15020665

**Published:** 2022-01-16

**Authors:** Riccardo Cacocciola, Badreddine Ratni, Nicolas Mielec, Emmanuel Mimoun, Shah Nawaz Burokur

**Affiliations:** 1 LEME, UPL, Univ Paris Nanterre, F92410 Ville d’Avray, France; beratni@parisnanterre.fr; 2 Saint-Gobain Research Paris, 93300 Aubervilliers, France; Nicolas.Mielec@saint-gobain.com (N.M.); Emmanuel.Mimoun@saint-gobain.com (E.M.)

**Keywords:** radome, scattering reduction, metasurface

## Abstract

A high-index dielectric radome seam is camouflaged with respect to a low-index dielectric radome panel by tuning the seam with carefully engineered metasurfaces. A transmission-line approach is used to model the metasurface-tuned seam and analytically retrieve the corresponding surface impedance, from which the unit-cell design is then tailored. Full-wave simulations and microwave antenna measurements performed on a proof-of-concept prototype validate the undesired scattering suppression effect in the case of normally and obliquely incident transverse electric and transverse magnetic wave illuminations. Robustness of the proposed solution to fabrication tolerances is also reported. The study presents metasurface-tuning as an easily implementable, frequency adjustable, and polarization insensitive solution to reduce the scattering of dielectric mechanical seams and improve the overall transparency performance of radome structures.

## 1. Introduction

Electromagnetically transparent materials, such as low-index foams or judiciously designed dielectric multilayer structures, are generally required in radio frequency (RF) systems. While ideal to achieve high RF performances, these materials often perform poorly from a mechanical standpoint and the use of high-index and hence low transparent dielectric pieces is then necessary to ensure the mechanical stability in such complex structures. Dielectric mechanical pieces are generally not optimized to be transparent at the operating frequency of a system and therefore, constitute a primary source of signal losses due to scattering phenomena. A good trade-off between RF transparent and structurally robust materials is then highly desirable. This is often the case in antenna radomes [1]. Large ground-based radomes are usually made of low-index dielectric or multilayer dielectric panels interconnected by high-index dielectric seams. While the panels are optimized to be transparent at the operation frequency of the antenna system enclosed in the radome, it is not the case for the mechanical seams, whose scattering signature is clearly visible in the far-field radiation patterns of the antenna. As such, mechanical seams behave as dielectric parasitic scatterers in the radome and constitute the primary source of signal losses and hence, radiation pattern degradation.

A well-known technique to reduce the scattering of radome seams is to integrate metallic structures, such as wires or grids, within the volume of the dielectric seam, which is then said to be *tuned* [1,2,3,4]. The scattering reduction induced by these metallic structures can be explained in terms of internal current redistribution phenomena within the dielectric volume [1]. When the high-index dielectric seam is excited by an impinging electromagnetic wave, the dielectric molecules of the object are polarized and give rise to polarization currents, from which the spurious scattering arises. When the dielectric volume is tuned with metallic wires or grids, additional currents are induced along these metallic structures. The polarization currents of the dielectric volume and the additional induced currents can then interfere constructively, resulting in an increase of the scattering, or destructively, reducing the overall scattering. When the currents induced on the metallic structures counterbalance the polarization currents of the dielectric, the scattering is suppressed. While wire- and grid-tuning solutions are commonly used to compensate the influence of these electromagnetically parasitic radome seams, they also present some important limitations. Firstly, for the scattering reduction phenomenon to be effective, the electric field of the impinging illumination should be aligned with the metallic structures integrated into the dielectric volume. Practically, this implies that the scattering will only be reduced for a specific polarization state of the impinging illumination. This is the case of wire-tuned seams where the use of grids instead of wires can alleviate this problem for a dual-polarization operation. A simple strategy to design grids for seam tuning applications is presented in [3]. A grid-tuned seam, consisting of two high-index dielectric layers sandwiching the grid, is modeled with transmission lines and the design of the grid is derived by equalizing the insertion phase delay of the tuned seam to that of a reference low-index dielectric material panel. While the analytical approach is straightforward, finding a grid geometry exhibiting the required behavior simultaneously for transverse electric (TE) and transverse magnetic (TM) polarizations mainly relies on a trial-and-error procedure. Furthermore, such solution may not be suitable for obliquely incident wave illuminations. A frequency-adjustable, polarization insensitive and oblique-incidence-robust alternative solution for seam tuning is therefore highly desirable.

Metasurfaces (MSs) are electrically thin periodic composite structures, which can be fully dielectric or metal-dielectric, and can be modeled as zero-thickness engineerable sheets to control the behavior of impinging electromagnetic radiations at will [5,6,7,8]. The metasurface’s unit-cell, also referred to as meta-atom, can be carefully designed to obtain specific amplitude and phase profiles, thus enabling a plethora of applications, ranging from polarization converters [9,10] to absorbers [11,12,13,14,15], as well as wide-angle impedance matching structures [16,17], imaging holography [18,19,20], complex waves generation [21] and many more [22]. More recently, programmable metasurfaces have attracted huge interests due to their versatility in performing different functionalities [23]. Self-adaptively reprogrammable functionalities without human participation have been fulfilled by a metasurface by integrating an unmanned sensing feedback system [24]. The development of artificial intelligence (AI) has also changed the manner to design metasurfaces [25]. As such, various applications, such as imagers [26] and recognizers [27] have been validated. In antenna related applications, metasurfaces are widely proposed for different functionalities such as beam forming, gain enhancement, profile reduction, decoupling, and so on [5,28,29,30,31,32,33]. Furthermore, a wide variety of theoretically presented and experimentally validated studies has solidified the role of metasurfaces in the field of electromagnetic illusion and invisibility cloaking [34,35,36,37,38,39]. Scattering cancellation solutions [34,40], such as plasmonic cloaking and mantle cloaking, allow to suppress the dominant scattering harmonics of the expanded scattered field of a scatterer by enveloping it in a judiciously designed cloak. By suppressing its scattering signature, the cloaked scatterer is then undetectable, in other words “invisible” to an observer. Such solutions, while suitable to cloak electrically small objects, are not well suited to efficiently suppress the scattering signature of an electrically large structure such as a high-index radome seam. In fact, as the scatterer grows in size, so does the number of scattering harmonics to be suppressed by the cloak, largely complexifying the required number of layers and the design of the cloak [34].

In this study, we aim to suppress the scattering signature of an electrically large, high-index dielectric seam that is used to interconnect two low-index material radome panels. A preliminary theoretical study based on a transmission-line approach has been recently proposed for the parasitic scattering suppression of a high-index seam [41]. The electromagnetic behavior of the seam is carefully tuned via judiciously engineered metasurfaces buried in the seam with respect to that of a low-index dielectric radome panel. The transmission-line model allows us to model the MS-tuned seam, retrieve the surface impedance of the buried MSs and deduce the meta-atom design. Following the theoretical study in Reference [41], a complete case study in which a high-index radome seam is camouflaged with respect to a low-index reference radome panel, is analytically studied and experimentally validated in the X-band at around 10.5 GHz. Full-wave simulations are presented and a proof-of-concept prototype is fabricated and measured in an anechoic chamber in the case of normally and obliquely incident TE- and TM-polarized illuminations. Moreover, experiments on slight variations of the prototype are also presented to study the fabrication tolerance of the camouflaging solution. Such alternative solution for camouflaging radome seams allows us to efficiently suppress the scattering signature of the seam, all while being polarization-insensitive, frequency-adjustable, and robust to oblique illuminations and fabrication tolerances.

## 2. Metasurface Design

Suppressing the scattering of a high-index dielectric seam can be achieved by integrating metallic structures into the seam volume [1]. When the currents induced on the metallic structures fully counterbalance the polarization currents of the dielectric seam, its scattering signature is eliminated. Moreover, by judiciously designing the tuning structures integrated in the dielectric volume, the scattering behavior of the high-index seam can be controlled to mimic the one of a low-index dielectric reference. In other words, a weakly transparent high-index dielectric radome seam can be camouflaged with respect to a highly transparent low-index dielectric radome panel, by carefully tuning it with buried metallic structures. In this study, we aim to camouflage an electrically large, dielectric radome seam (*ε_r_* = 3.0) with respect to a dielectric radome panel (*ε_r_* = 1.2), by tuning it with two metasurfaces.

**Figure 1:**
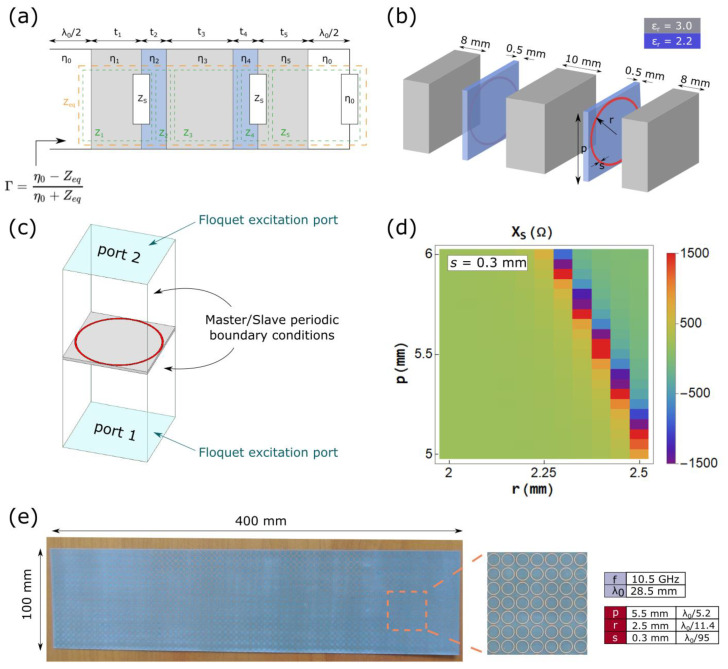
(**a**) Transmission line model of the metasurface-tuned seam. (**b**) Exploded view of a unit cell of the metasurface-tuned seam, comprising of three dielectric slabs (*ε_r_* = 3.0) sandwiching two dielectric substrates (*ε_r_* = 2.2 and tan δ = 0.001) onto which the meta-atoms are patterned. The ring meta-atom is characterized by its period p, outer radius r and width s. (**c**) Meta-atom’s simulation setup. The ring meta-atom is simulated in a periodic environment using Master/Slave boundary conditions and excited by Floquet ports. (**d**) Evolution of the reactance of a circular ring unit-cell as a function of its period p and outer radius r at 10.5 GHz. (**e**) Photography of the fabricated metasurface using printed circuit board technique with a zoom view on the unit-cells and a table giving the optimized unit-cell parameters.

The tuned seam is modeled by transmission line approach as proposed in Reference [41] and shown in Figure 1a. An exploded view of a unit cell of the metasurface-tuned seam is shown in Figure 1b. The seam consists of three dielectric slabs of relative permittivity *ε_r_* = 3.0 and thickness 8, 10, and 8 mm, respectively, sandwiching two 0.5-mm thick metasurfaces composed of dielectric substrates (*ε_r_* = 2.2) onto which the meta-atoms are patterned. It should be noted that the two metasurfaces are placed on the face of the substrate pointing outwards. Our goal is to carefully design the two buried metasurfaces so that the scattering behavior of the MS-tuned seam mimics that of an equally thick low-index dielectric (*ε_r_* = 1.2) radome panel at an operating frequency of 10.5 GHz (*λ*_0_ = 28.6 mm). At this operating frequency, the seam’s thickness is of the order of the wavelength (27 mm = 0.945 *λ*_0_). The material parameters and thicknesses chosen in this study correspond to commercially available dielectric slabs and the thickness of the tuned structure is close to the one of a commonly used radome seam. Moreover, real and lossy materials are considered throughout the study.

The MSs are treated as zero-thickness lumped shunt impedances *Z_S_* and the different regions defined by the dielectric slabs and the substrates as transmission line segments. In the model, *η*_0_ is the free-space impedance and *η_i_* the intrinsic impedance of the medium *i* of relative dielectric constant *ε_ri_*, defined as $\eta_{i}=\eta_{0}/\sqrt{\varepsilon_{ri}}$. The lengths *t_i_* of the transmission line segments correspond to the thicknesses of the different materials (8, 10, and 0.5 mm), as detailed in Figure 1b. Each segment is treated as a separate impedance (*Z*_1_ to *Z*_5_ in green in the model) and the full tuned structure can be described via an equivalent impedance *Z_eq_*, which is a function of the unknown surface impedances *Z_S_* of the metasurfaces. The reflection coefficient Γ at the input of the transmission line can then be written in terms of the equivalent impedance *Z_eq_* as:

(1) $$\begin{array}{c} \Gamma =\frac{\eta_{0}-Z_{eq}}{\eta_{0}+Z_{eq}}=\Gamma_{ref} \end{array}$$

with $\frac{1}{Z_{eq}}=\overset{i=5}{\underset{i=1}{\sum }}\frac{1}{Z_{i}}$ where $Z_{i}=\{\begin{array}{c} \eta_{i}\frac{Z_{S}+j\eta_{i}\tan\left(\frac{2\pi }{\lambda_{0}}\sqrt{\epsilon_{ri}}t_{i}\right)}{\eta_{i}+jZ_{S}\tan\left(\frac{2\pi }{\lambda_{0}}\sqrt{\epsilon_{ri}}t_{i}\right)}\;for\;i=1,\;4\\ \eta_{i}\frac{\eta_{i+1}+j\eta_{i}\tan\left(\frac{2\pi }{\lambda_{0}}\sqrt{\epsilon_{ri}}t_{i}\right)}{\eta_{i}+j\eta_{i+1}\tan\left(\frac{2\pi }{\lambda_{0}}\sqrt{\epsilon_{ri}}t_{i}\right)}\;for\;i=2,\;3\\ \eta_{i}\frac{\eta_{0}+j\eta_{i}\tan\left(\frac{2\pi }{\lambda_{0}}\sqrt{\epsilon_{ri}}t_{i}\right)}{\eta_{i}+j\eta_{0}\tan\left(\frac{2\pi }{\lambda_{0}}\sqrt{\epsilon_{ri}}t_{i}\right)}\;for\;i=5 \end{array}$

Since the main goal is to camouflage the seam with respect to the reference panel, the impedance of the two structures are matched by equalizing their respective reflection coefficients (Γ and Γ*_ref_*). This step allows us to analytically retrieve the value of the surface impedance *Z_S_*, the only unknown parameter of our model.

A sample of the low-index dielectric reference panel (*ε_r_* = 1.2) is measured in a WR90 waveguide (8.2 to 12.4 GHz) and a value of Γ*_ref_* = −0.0576 − j0.2046 is retrieved at 10.5 GHz. The surface impedance *Z_S_* is then analytically retrieved from Γ_ref_ according to Equation (1). A value of *Z_S_* = −1.7 × 10^−4^ − j41.8 Ω is thus obtained. The real part of the impedance is neglected due to its small value and the MS is approximated as purely reactive. A design of the MS unit cell can then be chosen based on the value of *Z_S_*. A circular ring meta-atom, whose design is presented in Figure 1b, is chosen for this study. The ring is characterized by the period p, the outer radius r and the ring’s width s. The ring meta-atom is simulated with the high frequency structure simulator (HFSS) commercially available code by Ansys [42]. The meta-atom is placed in an air box with Master/Slave periodic boundary conditions applied on the four lateral walls and excited from top and bottom by Floquet ports, as shown in Figure 1c. The lossy dielectric substrate (*ε_r_* = 2.2 and tan δ = 0.001) is also considered at this stage. Perfect electric conductor (PEC) boundary condition is applied to the ring to consider its metal characteristic. The evolution of the impedance’s reactance X_S_ as a function of p and r for a fixed value of *s* = 0.3 mm at 10.5 GHz, obtained by simulating the ring in the environment and illustrated in Figure 1c, is reported in Figure 1d. From this chart, the geometrical parameters required to achieve the desired *Z_S_* can be obtained. However, an optimization step is necessary to fine-tune the geometric parameters of the meta-atom and achieve the desired camouflaging effect in order to consider the finite lateral size of the structure that is not considered in the model. Finally, two MSs are fabricated and a complete proof-of-concept MS-tuned seam is realized. A picture of the fabricated MS, with optimized geometric parameters (p, r, s) = (5.5, 2.5, 0.3) mm = (λ_0_/5.2, λ_0_/11.4, λ_0_/95.2) at 10.5 GHz, is shown in Figure 1e. The MS is realized using standard printed circuit board (PCB) technology, where a 0.5-mm thick single copper-cladded F_4_BM220 Teflon woven glass fabric substrate having relative permittivity *ε_r_* = 2.2 and loss tangent tan δ = 0.001 is used.

**Figure 2:**
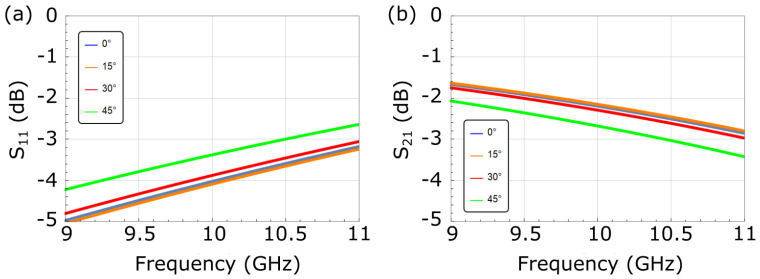
Evolution of the S-parameters of the optimized unit cell as a function of frequency for 0°, 15°, 30°, and 45° obliquely incident illuminations. (**a**) S_11_ and (**b**) S_21_. The S-parameters are obtained by simulating the optimized unit cell with Floquet ports and periodic boundary conditions.

The robustness of the optimized unit cell to obliquely incident illuminations is then assessed by studying the evolution of its reflection (S_11_) and transmission (S_21_) coefficients. The S-parameters are retrieved by simulating the optimized unit cell in the environment shown in Figure 1c. In this case, the Floquet ports are set such that the wave vectors of the incident plane waves are oriented 0°, 15°, 30°, and 45°, respectively, with respect to the normal of the meta-atom. The S_11_ and S_21_ coefficient is reported in Figure 2a,b, respectively. The overall stability of the S-parameters suggests that the unit cell is robust to obliquely incident illuminations and therefore, a good candidate to achieve a scattering reduction effect robust to changes in the direction of the incident wave.

## 3. Numerical and Experimental Validation

### 3.1. Simulated Scattered Field Distributions

**Figure 3:**
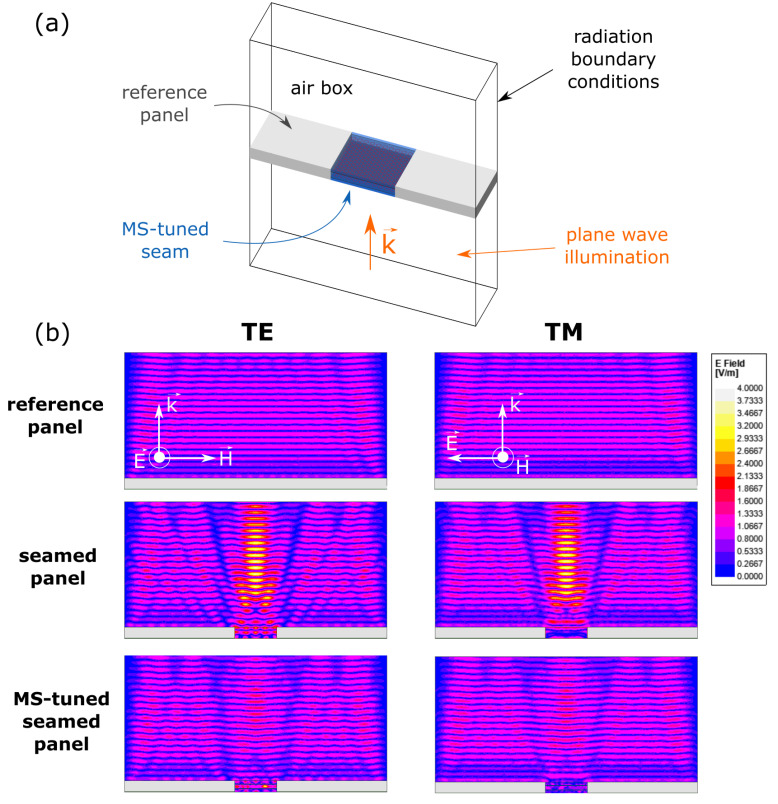
Numerical validation of the camouflaging effect. (**a**) Simulation setup, where a plane wave is used to illuminate the structure. (**b**) Scattered field distributions in the case of normally incident TE- and TM-polarized plane waves illuminating the reference panel (**first row**), the non-tuned seamed panel (**second row**) and the MS-tuned seamed panel (**third row**).

Once the MS design is engineered, the scattering reduction effect can be observed by comparing the scattered field distributions of the following three structures: the highly transparent low-index reference panel, the non-tuned seamed panel (without MSs), and the MS-tuned seamed panel. The seamed structures consist of two pieces of the reference panel connected via a tuned or non-tuned seam (with or without MSs, respectively). The simulation setup for the MS-tuned seamed panel is shown in Figure 3a. The structure is placed in an air box with radiation boundary conditions applied to the six faces and illuminated by a plane wave. Similar configuration is used for the simulation of the reference panel alone and for the seamed panel. The three structures are simulated in this radiation environment in the case of normally incident TE- and TM-polarized plane waves at 10.5 GHz and the scattered field distributions of the different structures are presented in Figure 3b. The direction of the plane wave radiation is shown by the wave vector k in Figure 3a,b. The scattered field is here obtained by subtracting the illuminating field from the total field. The perturbation induced by the non-tuned seam is visible for both polarizations in the scattered field distributions (second row) as a considerable increase in the scattered field amplitude and a disruption of the scattered planar wave fronts. This is often referred to as the forward scattering of the seam and is the scattering signature we aim to suppress. After tuning the seam with MSs (third row), the scattering signature of the seam is clearly suppressed and a regular scattered wavefront is restored. Since the presence of the seam is now nearly undetectable with respect to the bare panel, these simulation results suggest that the seam has been correctly camouflaged with respect to the panel.

### 3.2. Experimental Measurement of Far-Field Radiation Patterns

**Figure 4:**
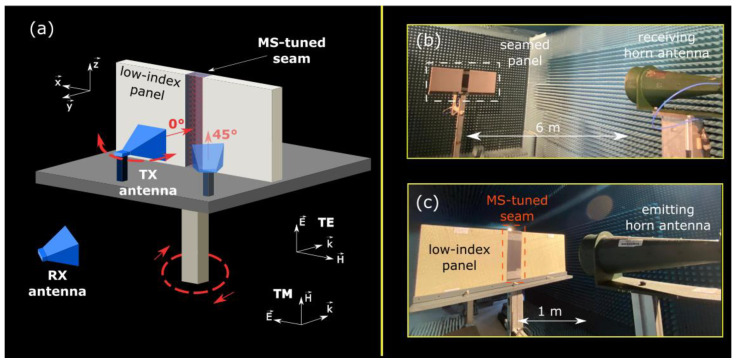
(**a**) Schematic illustration of the studied structure. Two low-index radome panels are connected via a high-index MS-tuned seam. The structure is illuminated by an emitting (Tx) antenna and a receiving (Rx) antenna allows us to measure the antenna radiation patterns for different angles of incidence. (**b**) Photography of the far-field measurement setup in an anechoic chamber. (**c**) Close up of the emitting antenna illuminating the seamed panel.

In order to further validate the proposed concept in a realistic far-field scenario, a proof-of-concept seam prototype is fabricated and measured in an anechoic chamber. The MS-tuned seam prototype consists of three dielectric slabs sandwiching two MSs patterned onto dielectric substrates, as shown in the exploded view of Figure 1b. The MS-tuned seam is 100-mm wide, 400-mm high, and 27-mm thick and connects two 450-mm wide pieces of a low-index dielectric radome panel of same height and thickness, as presented in Figure 4a.

The MS-tuned seam is realized by gluing three dielectric slabs and the two MSs, according to the illustration shown by the exploded view in Figure 1b. The microwave measurement consists of the seamed panel (two panel pieces connected via the seam) with an emitting (Tx) antenna mounted on an arm facing the seamed panel, as shown in Figure 4c, both mounted on a 360° rotating structure, and a receiving (Rx) antenna used to measure the radiation patterns (Figure 4b). Both antennas can operate over a wide frequency range spanning from 2 to 18 GHz. The seamed panel can be easily rotated with respect to the emitting antenna (Figure 4a) to study the impact of the seam in the case of obliquely incident illumination.

**Figure 5:**
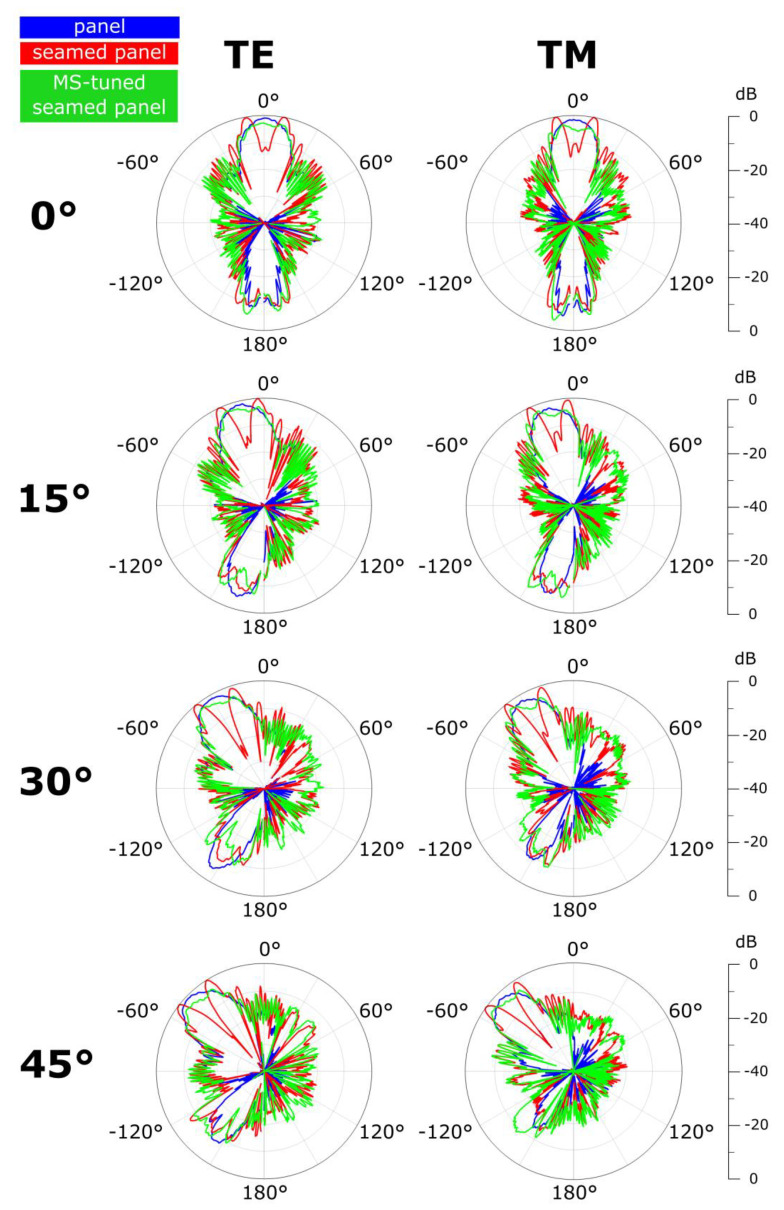
Measured far-field radiation patterns in the case of the reference panel (blue), the seamed panel without MSs (red) and the MS-tuned seamed panel (green), for 0°-, 15°-, 30°-, and 45°-incident, TE- (**first column**) and TM-polarized (**second column**) illuminations at 10.5 GHz.

Similar to the simulation approach presented above, a non-seamed piece of panel (our reference) as well as a non-tuned seamed panel (including two panel pieces connected via a non-tuned seam) are also measured. Far-field radiation patterns are then measured in the case of normally and obliquely (15°, 30°, and 45°) TE- and TM-polarized incident illuminations for the three structures and are reported in Figure 5 for a 10.5-GHz frequency. The radiation patterns for the three structures have been normalized with respect to the non-tuned seamed panel case. The presence of the non-tuned radome seam is clearly visible across the different cases (red patterns) as two lobes diverging the incident energy away from the desired angular direction. This constitutes the scattering signature of the seam, negatively affecting the radiation pattern of the Tx antenna in front of the radome panel (blue patterns). After tuning the seam with MSs (green patterns), the scattering signature is suppressed and the main lobe of the reference case is clearly restored. The radiation pattern restoration phenomenon can be observed for both TE- and TM-polarized illuminations for the entire 0°–45° incidence range, thus validating the polarization insensitivity and oblique incidence robustness of the proposed solution. The angular stability of the scattering reduction effect induced by the MSs is in good agreement with the stability of the S-parameters of the MSs unit cell, presented in Figure 2a,b.

### 3.3. Fabrication Tolerances

**Figure 6:**
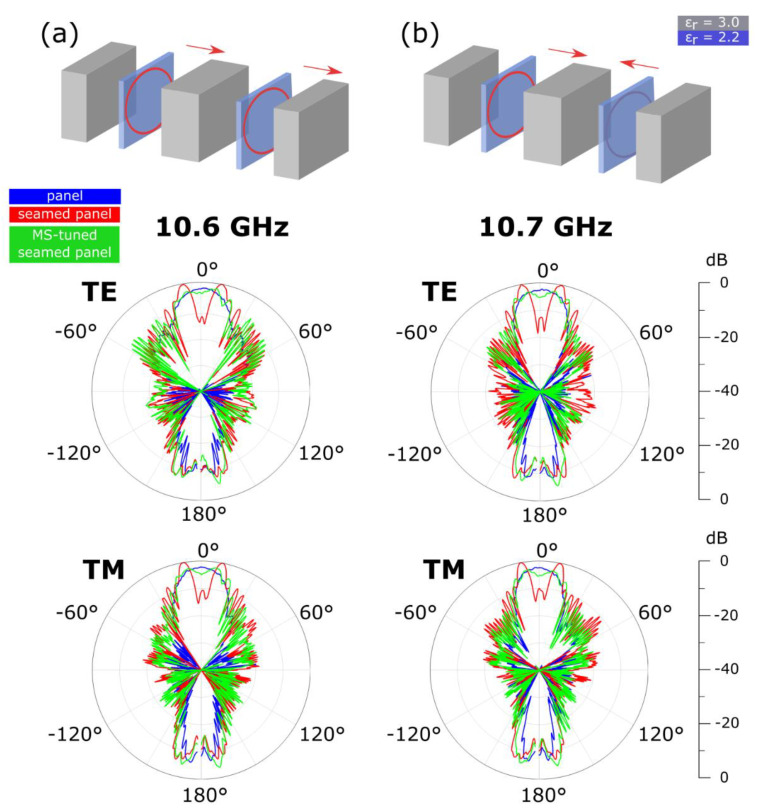
Variations of MS-tuned seam configuration and respective far-field radiation patterns. The red arrows in the schematic indicate the side of the substrate onto which the MS is patterned. (**a**) First variation: the first MS point inward and the second one outward. The scattering reduction effect is achieved at 10.6 GHz. (**b**) Second variation: both MS point inward. The scattering reduction effect is achieved at 10.7 GHz.

Finally, we study the robustness of the camouflaging effect of the MS-tuned seam to fabrication tolerances. In particular, the impact of the position of the MSs within the dielectric volume on the scattering reduction effect is assessed. In the original seam design (Figure 1b), the two MSs are patterned on the face of the substrate pointing outwards, thus at a burial depth of 8 mm. By manually changing the side of the patterned substrates within the seam, the burial depth of the MSs can be slightly changed. Two MS-tuned seam variations are obtained and reported in Figure 6. The red arrows indicate the face of the substrate onto which the MS is patterned. In the first variation (Figure 6a), one MS points inward (burial depth of 8.5 mm) while the other one outward (burial depth of 8 mm). In the second variation (Figure 6b), both MSs point inward (burial depth of 8.5 mm for both MSs).

The MS-tuned seam variations were characterized in the same far-field measurement setup described above. While the scattering reduction effect induced by the buried MSs is still observed in both variations for TE- and TM-polarized illuminations, a shift in the camouflaging frequency can be noted. In the case of the first variation, increasing the burial depth of one of the two MS of 0.5 mm induces a 100-MHz blueshift of the camouflaging frequency from 10.5 to 10.6 GHz. In the case of the second variation, adding 0.5 mm to the burial depths of both MSs induces a 200 MHz-blueshift from 10.5 to 10.7 GHz. In other words, for a given MS design, the deeper the MSs are located within the dielectric volume, the higher the camouflaging frequency will be. These results suggest a strong correlation between the position of the MSs within the volume of the seam and the frequency at which the scattering suppression is the most effective. Furthermore, since the burial depth of the MSs can be easily controlled during the fabrication process of the MS-tuned seam, it can be used as an additional parameter to fine tune the camouflaging frequency of the seam or easily adjust an already existing MS design to a slightly higher or lower frequency without the need to redesign the MS unit cell. Finally, the far-field results on the seam variations confirm the robustness of the proposed solution to fabrication tolerances.

## 4. Conclusions

In this study, a high-index radome seam has been camouflaged with respect to a low-index radome panel by burying carefully engineered MSs within the seam’s volume. The scattering signature of the seam, perturbing the radiation patterns of the antenna enclosed in the radome, arises from polarization currents within the dielectric volume. When the seam is tuned with MSs, the currents induced on their metallic elements can counterbalance the polarization currents and induce a controllable scattering reduction effect. By judiciously tuning the geometric parameters of the constituting unit cells of the buried metasurfaces, the scattering behavior of the MS-tuned seam can be controlled at will and mimic the one of a low-index reference radome panel. A transmission-line approach is exploited to model the MS-tuned seam and analytically retrieve the value of the MSs surface impedance, from which a meta-atom design can be deduced. The scattering reduction effect induced by the MSs has been simulated in a full-wave environment and experimentally validated with microwave anechoic chamber measurements on a proof-of-concept MS-tuned seam prototype. Far-field radiation patterns for a reference radome panel, a non-tuned seamed panel and a MS-tuned seamed panel have been reported at 10.5 GHz, validating the camouflaging effect for normally and obliquely incident TE- and TM-polarized illuminations. Robustness to fabrication tolerances of the proposed solution has also been validated by measuring two variations of the proposed MS-tuned seam design.

Metasurface-tuning of radome seams is a frequency-adjustable, polarization-insensitive alternative solution to drastically reduce the parasitic impact of seams on the radome’s transparency performance. While the scattering reduction effect still remains narrowband due to the resonant characteristics of the meta-atoms, the MS design can be easily adjusted with respect to the operating frequency of the radome. MS-tuning is particularly ideal for large ground-based seamed radomes working at fixed specific frequencies. Finally, the relatively low cost, easy implementation and scalability of the proposed MS-tuned seam design make it an attractive solution to improve the performance of seamed radomes.

## Data Availability

The data that support the findings of this study are available within the article.
